# Commencements

**Published:** 1899-06

**Authors:** 


### COMMENCEMENTS.

### University of California—College of Dentistry.

The commencement exercises of the College of Dentistry, University of California, were held at Berkely on Wednesday, May 17, 1899, at 10 A.M. Graduates:

| | | |
|---|---|---|
| William R Allin | George W Gove | Charles L Reich |
| Ricardo Arroyo | Frederick T Grant | Wallace H Renwick |
| William R Bacon | Francis J Gruss | Joseph P River |
| Arthur W Baker | Benagah R Hamlin | Maurice Schiller |
| Joseph Barnett | Leonore F Hermann | Thomas U Smyth |
| Robert J Blake | Thomas R Jones | Stephen S Southworth |
| May Blossom | Charles F Kuster | Abraham S Sullivan |
| Walter J Burridge | William J Lawson | Alonzo W Tate |
| Monroe N Callender | George W Likens | Howard A Tennyson |
| John A Colegrove | William H Mayhew | Rosa E Turner |
| Anna B P Croall | James B F Millar | Arthur H Wanz |
| Palmer H Dunbar | Edward M Mulrenin | William L Warnekros |
| Norman S Fairweather | Louis H Parks | Arthur L White |
| Cecil A Fugler | Andrew D Patterson | Edward O Whitney |
| Lee R Gambitz | Stephen L Piper | John J Williams |

Total 45.

### Northwestern University Dental School.

The annual commencement exercises of the Northwestern University Dental School were held in the Grand Opera House, Chicago, Ill., on Thursday afternoon, April 6, 1899.

The doctorate address was delivered by Alton H. Thompson, D.D.S., the salutatory by Thomas Reed, D.D.S., and the valedictory by Loran P. Akers, D.D.S.

The number of matriculates for the session was 587.

The degree of D.D.S. was conferred on the following graduates by Henry W. Rogers, LL.D., president of the university:

| NAME. | RESIDENCE. |
|---|---|
| Francis D Arter | Illinois |
| Thomas W Axtell | Iowa |
| Loran P Akers | Illinois |
| W H Albright | South Dakota |
| F S Anable | Minnesota |
| Sylvester W Arthur | Iowa |
| George M Brosnihan | Illinois |
| Wm H Baker | England |
| Arthur H Brown | Illinois |
| Chris Bostlemann | Illinois |
| Leslie O Barnard | Iowa |
| Louis Bloon | Illinois |
| Walter C Barber | Illinois |
| Howard T Bond | Illinois |
| Roscoe S Bayne | Illinois |
| Irying C Bronson | Iowa |
| L St C Bugbee | Wisconsin |
| Wyland C Bradshaw | Iowa |
| Carl H Brown | Illinois |
| William H Barth | Wisconsin |
| William E Bartels | Wisconsin |
| Harry B Bishopp | Illinois |
| James H Calder | Iowa |
| Robert L Cosner | Nebraska |
| Albert S Cork | Illinois |
| Walter DeW Cornell | Illinois |
| Frank E Carmichael | Nebraska |
| Herbert M Craig | Pennsylvania |
| Henry H Chilson | Wisconsin |
| C C Castle | Illinois |
| Chas B Campbell | Illinois |
| A E Childs | Iowa |
| Cara E Duth | Illinois |
| George L Dent | Illinois |
| Leroy Delibac | Illinois |
| Lewis C Dow | Indiana |
| A C Davidson | Iowa |
| Claude W Dodge | Nebraska |
| Wells Dewell | Iowa |
| Allen A Doughty | Iowa |
| Armin J Dieckmann | Michigan |
| Joseph W Dunning | Washington |
| Howard G Davis | Illinois |
| Gail B Elliott | Illinois |
| F F Ehlers | Iowa |
| A R Ebenreiter | Wisconsin |
| Arch E Ely | Missouri |
| Herman G Eakins | Michigan |
| Maria T Foster | Wisconsin |
| James E Forsyth | Australia |
| John S French | North Dakota |
| Walter P Flynn | Nebraska |
| Frank T Graham | Nebraska |
| Oren E Ganoe | Iowa |
| Frank T Gerecke | Nevada |
| Fred W Gethro | Illinois |
| Barney F Greenhow | Illinois |
| F R Grigsby | Illinois |
| Edward A Gibbon | Iowa |
| Ralph S Graham | Illinois |
| Albert O Haviland | Kansas |
| H E Harrison | Missouri |
| John A Huff | Illinois |
| Wm R Hepburn | Wisconsin |
| Max R Harvey | Wisconsin |
| William S Hicks | Illinois |
| Herbert G Haesler | Iowa |
| Henry F Helms | Iowa |
| H J Hemminger | Indiana |
| Ralph Y Hunt | Washington |
| D D Hallenbeck | Illinois |
| Ralph Hepler | Illinois |
| Oliver Harstad | Iowa |
| A J Hodges | Oregon |
| Chas W Hickman | Illinois |
| Robert W Johnson | Michigan |
| Ane M Jorgensen | Wisconsin |
| Richard R Johnson | North Dakota |
| Burton C Johnson | Illinois |
| James M Jones | Louisiana |
| Israel S Kirkwood | Illinois |
| Birdine King | California |
| John B Kalusner | Nebraska |
| Edward Kramm | Illinois |
| John C Kinney | Illinois |
| Wm O King | Wisconsin |
| Walter P Kountz | Pennsylvania |
| Fred Kestley | Wisconsin |
| Paul P Lucke | Wisconsin |
| Emery M Lotts | Iowa |
| Arthur C LaTouche | Illinois |
| Stephen A Lyon | Kansas |
| Martin J Linderholm | Illinois |
| Emil R Luebbert | California |
| Glenn L Merritt | Iowa |
| Jean Moyer | Illinois |
| Wm I Maddock | Illinois |
| Joseph E Miner | Illinois |

| NAME. | RESIDENCE. | NAME. | RESIDENCE. |
|---|---|---|---|
| John C Meng | Wisconsin | Clair W Roberts | Iowa |
| Nicholas J Maun | Nebraska | Geo A Ryder | Iowa |
| Samuel A Marlow | Wisconsin | Augustus M Rowan | Wisconsin |
| Harry D Manger | Minnesota | Lawrence F Ray | Michigan |
| Henry A Meyer | Oregon | Priscilla H Stansell | Ohio |
| N B W McCartney | Illinois | Ada P Swaim | Iowa |
| Ernest F McCartney | Iowa | Ernest S Sprenger | Wisconsin |
| James H McKay | Iowa | Burleigh M Smith | Iowa |
| Wm C McWhethy | Illinois | Ira B Sellery | Ontario |
| Oscar C Nylund | Sweden | Arthur Selvsberg | Iowa |
| Sylvia M F Napper | Illinois | David A Smalley | Illinois |
| Chas L Nitchelm | Illinois | F. W Simmonds | Iowa |
| Wm R Neff | Illinois | Harry I Swigert | Illinois |
| David M Olkon | Russia | Wm O Talbot | Mississippi |
| Burt T Osher | South Dakota | Reuben C Traynham | Texas |
| Bertram Older | Wisconsin | David J Thorp | Missouri |
| Chas A Petre | Illinois | Geo D Upson | Illinois |
| John S Pennington | Iowa | Wm E Underwood | Wisconsin |
| R K Pinkerton | Illinois | H N Van Denbergh | Michigan |
| Fred W Parker | Illinois | Anthony J Williams | Wisconsin |
| Harry E Pike | Illinois | Chas E Winter | Illinois |
| Henry V Pfaff | Missouri | Nicholas P Webber | Iowa |
| Harry E Pier | Wisconsin | Eugene S Willard | Alaska |
| Walter J Petrie | Illinois | Ernest I Waldberg | Illinois |
| Wm J Prideaux | | Frances M Walsh | Illinois |
| Frank S Potts | Wisconsin | Chas F Wadsworth | Illinois |
| Albert B Potter | Kansas | Frank M Welch | Indiana |
| Chas J Reinfried | Iowa | David J Wright | Manitoba |
| Thomas Reid | Scotland | Samuel A Wright | Minnesota |
| Jas A Robertson | South Dakota | Chas A Walton | Illinois |
| Chas F Rounseville | Illinois | Macomb L Wright | Iowa |
| H C Rodenhouse | Illinois | Ernest H Wilson | South Dakota |
| Frank M Reed | Illinois | S W Zipperman | Russia |

Total, 162.

### Marion-Sims College of Medicine—Dental Department.

Commencement exercises Wednesday evening, April 19, 1899, at Fourteenth Street Theater. Graduates:

| NAME. | RESIDENCE. | NAME. | RESIDENCE. |
|---|---|---|---|
| Joseph M Bobbitt | Missouri | Augustus R Parrish | Missouri |
| Lewis A Chamberlin | Missouri | Benjamin L Renfrow | Illinois |
| Leander O Crall | Illinois | Harvey B Scarsdale | Illinois |
| Otto F H Depperman | Indiana | Joseph F Shiermann | Illinois |
| Amos Ditty | Illinois | John A H Schwind | Illinois |
| Arthur C Edmonds | Durban, S Africa | Robert L Shanklin | Illinois |
| Gustavus J Ellwanger | Missouri | Joseph J Simmons | Mississippi |
| Jonathan H Gallemore | Missouri | William W Von Wormer | Illinois |
| Oscar Hammer | Missouri | Richard E Vernon | Illinois |
| Alvin N Martin | Tennessee | Edmund L Watson | Arkansas |
| Leo G McKellops | Missouri | William A Weis | Missouri |
| David E Morrow | Missouri | Fielden W Wiseman | Missouri |
| Elmer L Page | Missouri | Joseph H Witfield | Missouri |

Total 26.

### Chicago College of Dental Surgery.

The seventeenth annual commencement exercises of the Chicago College of Dental Surgery (Dental Department of Lake Forest University) were held in Central Music Hall, Chicago, Ill., on Wednesday afternoon, April 5, 1899.

Addresses were delivered by Dr. John H. Finley, president of Knox College; Lewis Stewart, M.A., Ph.D., of Lake Forest University, and Rev. Wm. M. Lawrence, D.D.

The class valedictory was delivered by John P. Luthringer, D.D.S.

The number of matriculates for the session was 507.

The degree of D.D.S. was conferred on the following graduates by Truman W. Brophy, M.D., D.D.S., LL.D., president of the college:

Emil A Ableiter

Lewis J Andrews

Fred C Angle

Amos E Adsit

H W Boon

I A Burnett

John Bohr

Rudolph Beck

Roy G Booth

Albert J Buchheit

Otto Baumrucker

Geo E Bratten

John H Burton

Silas A Beason

A E Bartholomew

Geo B Brown

S A Brady

Geo P Brenner

C L Bartholomew

F E Blakeslee

Geo H Bomer

Edw G Burgman

Holmes G Brown

Orville L Bates

Edgar S Barnes

Clark M Cuthbert

William A Cox

Edgar D Chandler

Homer L Cheever

Carl B Case

Elbert H Cornwell

Wm L Colyer

Oscar J Cunningham

Geo W Diepenbrock

Wm Dorfner

Glenn S Dolson

Peter T Diamond

Abraham Drosdowitz

E G Dundass

Mell M Everett

Griffith D Evans

James W Ewin

John English, Jr

A J England

H M Ferguson

Emery L Fincham

Bruno W Fick

Lemuel P Fry

David W Fithian

Conrad H Forster

Howard Frace

C A Flemming

W G Fortune

James M Gardner

Edwin J Greenfield

Fred A Gray

Will M Gabriel

W P Gasser

Warren A Gamble

Chas E Gerretson

Edgar L Gausby

J J Gillane

Joseph W Hardin

Frank S Hanscomb

Fred L Hamil

Frank J Haessler

Ralph E Hayden

Frederick Hewetson

Robert C Horner

Wilfrid P Harvey

Edw J Hanan

Fred E Hall

Nazareth S Haradjian

Paul H Harlan

Harry C Higgins

James L Hamilton

Elton E Hankins

F H Helt

H. H. Herren

Otto Holinger

Virgil Hoffer

Chas E Hirth

John C Hothan

Thomas H Harris

Alexander Ivey, Jr

John E Isely

Geo T Jones

Edw E Johnson

John A Kaufer

Conrad G Kinstad

Geo W Kerner

Frank J Kuehn

J P Luthringer

L B Lindeboom

Walter M Long

Oscar A Laughlin

William Luxmore

Eric Lindholm

Geo R Lanning

Edwin H McTaggart

R D McKean

Richard J McClevy

Irvin E McVay

Hugh J MacKechnie

Richard D Moran

Fred B Moorehead

John McPhee

James M Manton

George Massart

Louis H Maas

Albert E Morey

Manly H Michaelis

Geo W Nevins

Richard E Nixon

Carl J Nielson

Emmet M O'Keefe

John Orth

William C Penrose

C G Pomainville

John H Pierce

Malcolm Pounder

Fred A Richards

Edw A Ritzenthaler

Geo M Ruttan

Robert M Riggs

Benj F Redman

Wm H Roth

Alfred E Rocke

G A Russell

Harry E Stevenson

Truman Shattuck

Clem E Shidler

Walter R Stokes

C A Spellman

Harry J Smith

Harry D Shaw

John J Seidscheck

Richard A Smith

Alva L Spindler

Edward H Steel

David L Stanton

Allison Smith

Geo Schneider

Walter R Shell

G W Torrey, M.D.

Berna S Tyler

Edward S Wingren

Dudley Welch

Chas A Wood

B. Williamson

Wm E Weis

James A Wells

J F F Waltz

John C Warnock

C W Wolfenberger

Wm S Walters

Chas A Wayde

Philip J Wendel

P M Wuillemin

S I Williams

Thos J Wilson

H E Walsh

John A Zartzin

Total, 163.

### Baltimore College of Dental Surgery.

The fifty-ninth annual commencement exercises of the Baltimore College of Dental Surgery were held in the Lyceum Theater, Baltimore, Md., on Friday evening, March 31, 1899.

The number of matriculates for the session was seventy-two.

The degree of D.D S. was conferred on the following graduates by Professor M. W. Foster, dean of the college:

| NAME. | RESIDENCE. | NAME. | RESIDENCE. |
|---|---|---|---|
| F S Adams | Kansas | L A Johnson | North Carolina |
| E B Alkire | West Virginia | S B Johnston, Jr | New Jersey |
| G W Atkinson | Massachusetts | E D Jacobus | New Jersey |
| C W Atterbury | Kansas | F H James | Pennsylvania |
| W W Barham | California | T D Kelley, Jr | Kentucky |
| W H Beach | Maryland | L R Lafond | Canada |
| R W Rickford | Maine | F H Lambert | Canada |
| W H H Bixler | Maryland | H M Leonard | Canada |
| G W Cheadle | Oregon | S P J Lee | North Carolina |
| A E Cary | Connecticut | R T Lakin | Maryland |
| L D Coriell | Maryland | A W Leard | Canada |
| A J Cohn | Louisiana | J S Lowther | Canada |
| W W Cushman | New Hampshire | G H Marven | New Brunswick |
| S J Craig | Pennsylvania | C S McArthur | Nova Scotia |
| G de Chiara | New York | M G Munro | Massachusetts |
| M Douglas | Canada | T E McCraith | New York |
| A G Duncan | Pennsylvania | A L Pendergrass | Arkansas |
| C B Dickson | Kentucky. | D Payne | Arkansas |
| Wm H Emmett | Connecticut | E G Price | New York |
| T O Flannagan | West Virginia | P R Stillman | New York |
| S Frontis | North Carolina | E B Sefton | Maryland |
| C A Gowans | Utah | Eva E Semon | Maryland |
| C E Gaston | Mississippi | C J Sellors | Pennsylvania |
| H C Gore | Maryland | H L Smyth | West Virginia |
| W E Gammans | Maine | F E Smallwood | Nova Scotia |
| A M German | New York | C R Stewart | West Virginia |
| J E Gordon | Kentucky | H H Streett | Maryland |
| C A Humphreys | New York | J E Taylor | South Carolina |
| B Holmboe | Norway | J P Taggart | Ohio |
| J W Halsell | Texas | C L Woodworth | Maine |
| H H Johnson | Nova Scotia | W N Weeks | North Carolina |

Total 62.

### Pennsylvania College of Dental Surgery.

The forty-third annual commencement exercises of the Pennsylvania College of Dental Surgery were held in the Academy of Music, Philadelphia, Pa., on Thursday evening, March 30, 1899.

The address to the graduates was delivered by Professor C. N. Peirce, D.D.S.

The number of matriculates for the session was three hundred and twenty-eight.

The degree of D.D.S. was conferred on the following graduates by I. Minis Hays, M.D., president of the college:

| NAME. | RESIDENCE. | NAME. | RESIDENCE. |
|---|---|---|---|
| George Algor | New Jersey | William D Knecht | New Jersey |
| James Agnew | Canada | Victor E Koch | Missouri |
| Sidney F Bridges | Canada | Julius A T Krieg | Germany |
| George N Bates | Ohio | John H Keiser | Pennsylvania |
| George C Bryant | Pennsylvania | Clara M Loetschert | Germany |
| Gilderoy O Burlew | New Jersey | Leigh A Langstroth | Canada |
| John Brandt | Minnesota | George W Laidlaw | Scotland |
| Lloyd D Bickley | Pennsylvania | Lemuel J Morgan | Pennsylvania |
| Thales D Bugbee | Vermont | F C Marggraff | Connecticut |
| John W Biddle | Pennsylvania | K C MacDonald | Canada |
| George W Boyd | Pennsylvania | William H Mohler | Pennsylvania |
| John D Benjamin | Pennsylvania | Roy D Marsh | New York |
| Ruth Bitting | Pennsylvania | Lester L McKay | Pennsylvania |
| Malcolm H Barr | Pennsylvania | Frank R Miller | California |
| Wilson W Bolton | Pennsylvania | J Preston Metzger | Pennsylvania |
| George R Cook | Pennsylvania | Frederick W Mace | Pennsylvania |
| Francis B Crane | Connecticut | Francis J Mahony | Massachusetts |
| Daniel R Crump | Pennsylvania | Justin B McCarthy | Jamaica |
| Fred W Carroll | Massachusetts | Charles P Moore | Canada |
| H H Cleveland | Massachusetts | John C Nugent | Pennsylvania |
| John C Curry | Pennsylvania | J Courtney Nedwill | Ireland |
| William M Chandler | Connecticut | Alice M Norton | Pennsylvania |
| Erwin E Connor | Massachusetts | Reuben R Picard | Pennsylvania |
| James E Collins | Quebec | Charles D Peterson | Pennsylvania |
| Henry B Campbell | New Jersey | John G Powell | Pennsylvania |
| Anna M Carstan | Germany | Walter C Richman | Pennsylvania |
| George R Doan | Pennsylvania | John H Ruth | Pennsylvania |
| Walter Daniel | Pennsylvania | Max Raff | Pennsylvania |
| Carl H DeLonge | Germany | Alfred C Russell | Canada |
| Charles H Dunham | New Jersey | B A Richardson, Jr | Indiana |
| Calvin A Davis | Pennsylvania | Franklin Rightmire | New Jersey |
| Albert N Drury | New York | Robert R Royster | Massachusetts |
| Hattie A Dean | New York | William E Richards | Pennsylvania |
| Fred'k J R Dean | New York | W Reeves Robinson | New Jersey |
| Leopold A DeRosa | Egypt | Thomas D Renfrow | Kentucky |
| Richard Ellis | New York | David F Strock | Ohio |
| Thomas J Evans | Pennsylvania | James F Shute | Canada |
| Emil Frey | Switzerland | George H Spieker | Pennsylvania |
| Richard M Flaherty | Pennsylvania | LeRoy D Shafer | Pennsylvania |
| William P Felch | New Jersey | Maurice E Sargent | New Hampshire |
| L I Fleming | Canada | Conrad Schroeder | Pennsylvania |
| Helen Fox | Russia | William M Stetler | Pennsylvania |
| Ami H Gunther | Ohio | Harry M Sober | Pennsylvania |
| Frank D Geer | Pennsylvania | S A Sturdevant, Jr | Pennsylvania |
| Robert E Grennan | Vermont | Thomas Thomson | Canada |
| Ralph S Hunt | Massachusetts | Edwin D Trimmer | New Jersey |
| Arthur G Hodge | New York | Guy C Tower | Massachusetts |
| William Hecht | Pennsylvania | Flora E Upson | Connecticut |
| E V Hendrixson, Jr | Pennsylvania | Frank T Waters | Massachusetts |
| Elizabeth Y Hood | New Jersey | Martin I Wesseler | Germany |
| Sophie Hullstrung | Germany | James E White | Canada |
| Fred H Johnson | Canada | Julia C Wood | Pennsylvania |
| Marks Kohn | Pennsylvania | Albert K Wood | New Jersey |
| Annette L Kowler | Bulgaria | J Finley Wark | New York |
| Charles E Kantz | Pennsylvania | Alexander W Watt | Canada |
| Sophia Korotkin | Russia | | |

Total 111.

### Philadelphia Dental College.

The thirty-sixth annual commencement exercises of the Philadelphia Dental College were held in the Academy of Music, Philadelphia, Pa., on Friday evening, April 7, 1899.

The address to the graduates was delivered by Professor

Henry C. Boenning, M.D., and the valedictory address by Charles N. Reinig, D.D.S.

The number of matriculates for the session was three hundred and thirty-four.

The degree of D.D.S. was conferred on the following graduates by ex-Governor James A. Beaver, president of the board of trustees:

| NAME. | RESIDENCE. |
|---|---|
| Edmund J Abbott | Connecticut |
| Alejandro Andrade | Colombia, S A |
| Walter K Ashton | Pennsylvania |
| Walter S Bahner | Pennsylvania |
| William Z Barrett | Ohio |
| Henry D Bedford | New York |
| Wm O Beecher | Connecticut |
| Joseph Birnbaum | Roumania |
| Oliver L Braud | Louisiana |
| Manuel J Brazill | Connecticut |
| J B Calvo | Colombia, S A |
| H L Chandler | Pennsylvania |
| A Chiavaro, M.D | Italy |
| Ira J Coe | New York |
| Johan Cohn | Roumania |
| Forrest A Cousins | Maine |
| Chas F Cornelius | New York |
| Edgar D Crawford | Pennsylvania |
| Frank L Davies | Maine |
| Edward H Derr | Pennsylvania |
| Bessie A Douglass | Pennsylvania |
| Clara A Edwardes | Jamaica |
| Chas B Fickes | Ohio |
| Henry G Fisher | Pennsylvania |
| Michael F Flynn | Massachusetts |
| Sheltan C Frederic | Alabama |
| C R Fundenberg | Pennsylvania |
| Wm J Galbraith | Canada |
| Louis E Gieser | Delaware |
| Wm C Ginter | Pennsylvania |
| Frank H Goddard | England |
| W S Goodwin | Canada |
| Chambille Gordon | West Indies |
| John J Gribbin | Washington |
| Geo H Griffith | New Jersey |
| Frank J Haas | Pennsylvania |
| Robert T Hall | California |
| Hugo Herz | Hungary |
| Ralph Herz | Hungary |
| Samuel S Hess | Pennsylvania |
| Isaac B Jacobs, 3d | Pennsylvania |
| Arthur V Jolliffe | Canada |
| James C Kelley | Massachusetts |
| B G Klinetob | Pennsylvania |
| J F Knowlton | Ohio |
| S L Landon | New Jersey |
| T L Larseneur | Canada |
| Harold Lawrence | Canada |
| Hardouin Lionais | Canada |
| R W MacDonald | Pennsylvania |
| Jno A MacNeil | Canada |
| W A MacNicholl | New York |
| John L Mansir | Massachusetts |
| Wilbur C Marsh | Pennsylvania |
| Geo A Martin | Canada |
| Dorothy Maryson | New York |
| Hubert F Milford | Australia |
| Edw H Morrison | California |
| S G Newcomb | New Jersey |
| E B Newell | Michigan |
| W I Northrop | Oregon |
| Grant W Osborne | Pennsylvania |
| Allie N Osgood | Maine |
| Harold C Parker | New York |
| Frank H Paul | Pennsylvania |
| Zotique J Payan | Rhode Island |
| Alexander Peacock | Canada |
| N V Pockley | Australia |
| B Frank Reade | Canada |
| Charles N Reinig | Montana |
| Conrado Rivera | Porto Rico |
| Charles E Rose | Pennsylvania |
| James T Savery | Pennsylvania |
| C C Schneider | Connecticut |
| Martin F Shannon | Pennsylvania |
| Wm C Sharkey | Pennsylvania |
| Walter B Shaw | Connecticut |
| Perry R Skinner | New York |
| Edw H Smith | Massachusetts |
| Edw R Smyth | Pennsylvania |
| Edw A Sprague | Maine |
| Emil E Steiner | Pennsylvania |
| Wm L Stevenson | Pennsylvania |
| Wm P Stone | Massachusetts |
| Edw D Stout | Pennsylvania |
| Charles H Tilton | New York |
| Orie O Tolles | Pennsylvania |
| Francis H Tomlin | New Jersey |
| R C Turner | New York |
| R M Van Dervoort | Colorado |
| Sidney V Vega | Louisiana |
| Lewis J Walker | Pennsylvania |
| Arthur W Walsh | Natal, So Africa |
| Charles F Wilbur | Connecticut |
| John B Winslow | Maine |
| E A Zoberbier | Germany |

Total 96.

### University of Maryland—Department of Dental Surgery.

The annual commencement exercises of the Dental Department of the University of Maryland were held in the Lyceum

Theater, Baltimore, Md., on Thursday evening, March 30, 1899.

The address to the graduates was delivered by Rev. Elbert S. Todd, D.D., and the class oration by Linnæus C. Shecut, D.D.S.

The number of matriculates for the session was two hundred and sixteen.

The degree of D.D.S. was conferred on the following graduates by Ferdinand J. S. Gorgas, M.D., D.D.S., dean of the Dental Department:

| NAME. | RESIDENCE. |
|---|---|
| C T Alexander | Georgia |
| James H Baker | North Carolina |
| M C Boyden | North Carolina |
| Charles J Bragdon | Maine |
| A Broecking | Germany |
| P Du B Brooker | South Carolina |
| Maughs M Brown | Mississippi |
| William S Brown | Maryland |
| Wilbur F Browne | Maine |
| John W Carlton | North Carolina |
| Franklin J Cline | Virginia |
| James R Copeland | South Carolina |
| W B Dailey, B.Ph | Ohio |
| Pleasant R Falls | North Carolina |
| T T Fauntleroy | Virginia |
| W A Fillmore | California |
| Edward A Gamard | Louisiana |
| F E Hammond | New York |
| W F Hammond | New Hampshire |
| Walter E Hankin | New York |
| Asa I Harris | Virginia |
| Harry G Hawley | New York |
| W C B Hoffman | North Carolina |
| David G Hollinger | Pennsylvania |
| Arthur N Hyde | New Jersey |
| Herman Igel | Germany |
| John N Johnson | North Carolina |
| George S Johnston | Canada |
| Arthur B Jones | Bermuda, W I |
| James G King | Virginia |
| Alfred J Kohly | Cuba |
| Cyrus Kurtz | Pennsylvania |
| W T Lineweaver | Virginia |
| William L Logan | Georgia |
| Louis M Lynch | Georgia |
| Stewart O Marshall | Kentucky |
| Samuel B Milford | Virginia |
| S B Moscovitz | New York |
| E V McConnell | Georgia |
| DeWitt C McIver | North Carolina |
| William R McLeod | South Carolina |
| Benjamin F Orr | Virginia |
| Charles E Outcalt | West Virginia |
| Raiman Petty | New York |
| James W Pitts | South Carolina |
| William R Pond | Vermont |
| Warren Price | Maryland |
| John A Roach, Jr | North Carolina |
| Robert J Scott, Jr | New York |
| Wallace P Scott | Maine |
| William T Shannon | Georgia |
| Linnæus C Shecut | South Carolina |
| Frank E Smith | New Brunswick |
| George C Smith | Maryland |
| Harold B Smith | New York |
| Elmer M Steele | Virginia |
| Harry C Stover | Pennsylvania |
| Mina F Styne | Virginia |
| Tameji Takashima | Japan |
| Willard H Tibbetts | New York |
| Herman Tropp | Russia |
| Adrew B Wardlaw | South Carolina |
| Leroy M White | New York |
| Joel Whitaker | North Carolina |
| William H Wise | Virginia |

Total 65.

### Cincinnati College of Dental Surgery.

The sixth annual commencement exercises of the Cincinnati College of Dental Surgery were held in the Auditorium, Cincinnati, Ohio, on Thursday evening, April 6, 1899.

An address was delivered by Professor G. S. Junkerman, M.D., D.D.S., dean, and the valedictory address was by Professor O. W. Martin, A.M.

The number of matriculates for the session was one hundred and nine.

The degree of D.D.S. was conferred on the following graduates by Mr. Francis B. James, president of the board of trustees:

| NAME. | RESIDENCE. | NAME. | RESIDENCE. |
|---|---|---|---|
| Fred C Ahlers | Ohio | Richard J Morris | Kentucky |
| And D Armstrong | Ohio | Joseph H Nuxoll | Kentucky |
| Arthur D Bowyer | Ohio | Chas W Owens | Indiana |
| T N Crowley, M D | Illinois | Wm H Palmer | Ohio |
| Orsa B Coxen | Indiana | Everett C Perry | Kentucky |
| Linus H French | Ohio | Homer D Rymer | West Virginia |
| Philip N Foley | Kentucky | Claire B Schleef | Missouri |
| B Frank Gray | Ohio | Milton A Smith | Indiana |
| Geo E Harper | Ohio | Arthur D Sloan | Ohio |
| Geo W Halley | Ohio | Walter J Teeters | Ohio |
| S K Hirshman | Ohio | Carl M Voelkel | Ohio |
| A L Hollowell | Ohio | Geo W Walter | Indiana |
| Walter A Holmes | Ohio | Alfred H Wagner | Ohio |
| James J Hill | West Virginia | J Herbert West | Ohio |
| Fred S Homann | Indiana | John J Walker | Ohio |
| Elijah P Houk | Indiana | Walter L Wright | Ohio |
| Wm C Johnson | Kentucky | Benj F Wade | Ohio |
| William E Knight | Ohio | Jas R Youngson | Ohio |

Total 36.

### Pittsburg Dental College.

The third annual commencement exercises of the Pittsburg Dental College were held in the Alvin Theater, Pittsburg, Pa., on Tuesday afternoon, April 4, 1899.

Addresses were delivered by Rev. J. Crocker White, D.D., and James F. Baldwin, A.M., M.D., of Columbus, O. The address to the graduates was made by Dr. J. G. Templeton, dean of the college.

The number of matriculates for the session was one hundred and ninety-six.

The degree of D.D.S. was conferred on the following graduates by Dr. W. J. Holland, chancellor of the university:

| | | |
|---|---|---|
| L E Ackerson | C S Horner | C C Rinehart |
| P A Ambrose | W J Holroyde | J K Scott |
| M J Barchfield | J N Kramer | H A Seitz |
| W C Butler | O M Kennedy | F A Schade |
| C R Blair | R E King | G L Stewart |
| F L Crow | W B Lytle | W H South |
| F N Cook | M R Meredith | C A Simpson |
| C B Dennis | W D Moore | J T Smith |
| A I DeRoy | L V Marquis | J R Titzel |
| A Deucker | C L McChesney | C W Thomas |
| W C Evans | J W McKee | W Throckmorton |
| C C Elliott | G W McKean | G A Urling |
| W D Fawcett | I D Orr | A E Wilhelm |
| Oscar Fausner | A H Pidgeon | C E Whitehead |
| D J Griffiths | C E Peters | E A Waugaman |
| E C Hawkins | T A Phillips | D N Williams |
| W G Hughes | S H Ralston | W J Walthour |
| T A Hogan | E J Roney | B E Wright |

Total 54.

### University of Buffalo.

The seventh annual commencement exercises of the Department of Dentistry of University of Buffalo were held in Music Hall, Tuesday evening, April 25, 1899. Graduates:

| | | |
|---|---|---|
| Frank Anderson | Charles E Gillam | George F Mouthrop |
| George M Austin | Gladstone Goode | Mansfield E Mooney |
| William G Beaumont | Bertrand O Harmon | Arthur B Muir |
| Charles A Bennett | Charles H Hickelton | Emanuel Muntz |
| Frank J Biker | Abram Hoffman | Daniel M Murray |
| Michael C Bradley | George L Horton | Thomas F O'Shea |
| William J Burke | Arthur F Isham | George O'Leary |
| Duncan A Cant | William D Jacob | Frederick W Orwan |
| Frederick W Champlin | Alfred O Jerrett | Herman E Reynolds |
| Charles H Churchill | Arthur J Jessel | Clare E Robinson |
| Arthur B Cobb | Clement D Kennedy | William J Roche |
| Frank W Cook | Burt Kinsella | Robert Sabin |
| Frederic S Cox | Francis M Lee | Robert R Schmidt |
| Leon Van Cursons | William N Leonard | Grace N Shirley, B. L. |
| Guy R Danforth | Harry H Luton | Howard A Smith |
| George E Dougall | Harry K Mason | Louis W Smith |
| John E Dunn | Russell G W Merkley | Alonzo W Tracy |
| Harry G Fairfield | Stanley A Merkley | James F Wardner |
| Robert J Fletcher | John Middaugh | William H Willson |
| Charles J Fraley | | |

Total 58.

### Southern Medical College—Dental Department.

The twelfth annual commencement exercises of the Dental Department of Southern Medical College were held in the Grand Opera House, Atlanta, Ga., on Friday evening, March 31, 1899.

The address to the graduates was delivered by Professor Charles Lane.

The degree of D.D.S. was conferred on the following graduates by Col. N. T. Hammond:

| NAME. | RESIDENCE. | NAME. | RESIDENCE. |
|---|---|---|---|
| O J Bender | North Carolina | C W McCalla | Georgia |
| G L Brown | Florida | B R McLaughlin | Georgia |
| T W Brock | Georgia | Wm McLeod | Georgia |
| E L Crabb | Cuba | J W McConnell | Georgia |
| C F Crouch | Georgia | J A Montgomery | Georgia |
| J B Donaldson | Georgia | A A Pfieffer | Alabama |
| C F Davis | Alabama | A T Reeves | Alabama |
| N Eisenmann | Louisiana | J Rogers | Texas |
| Love Felton | Georgia | J O Seamans | Georgia |
| T C Gibson | Georgia | D R Seastrunk | Texas |
| J R Henry | Louisiana | D E Shackelford | Alabama |
| Clifton Jones | South Carolina | J L Ragan | Louisiana |
| S A Lightfoot | Alabama | G M Terry | Texas |
| E E Lovalace | Georgia | T P Tison | Georgia |
| C Z McArthur | Georgia | Virgil Voorhis | Florida |
| C G Martin | Mississippi | B Wildauer | Georgia |

Total 32.

### Vanderbilt University, Department of Dentistry.

The twentieth annual commencement exercises of the Dental Department of Vanderbilt University were held in the Theater Vendome, Nashville, Tenn., on Thursday, March 30, 1899.

The address to the graduates was delivered by Rev. James I. Vance.

The number of matriculates for the session was 165.

The degree of D.D.S. was conferred on the following graduates by Vice-Chancellor Rev. W. F. Tillett:

| NAME. | RESIDENCE. | NAME. | RESIDENCE. |
|---|---|---|---|
| F L Bethancourt | Louisiana | J R Johnson | Alabama |
| C F Boyer | Mississippi | E E Kaiser | Tennessee |
| J J Boyd | Tennessee | G Montgomery | Tennessee |
| H G Bow | Kentucky | M McNeely | Tennessee |
| L B Duncan | Kentucky | Chas McKennon | Arkansas |
| J L Dunn | Alabama | Jas A Moore | Tennessee |
| W W Ezelle | Alabama | L B Price | Alabama |
| J H Elliott | Texas | J D Poindexter | Alabama |
| J R Eleazer | Tennessee | L J Ramage | Alabama |
| W M Fariss | Tennessee | E Riviere | Georgia |
| J E Frazer | Alabama | G W Reynolds | Arkansas |
| J L Griffin | Kentucky | Alex Sledge | Alabama |
| Will Gillespie | Tennessee | W P Sims | Tennessee |
| H T Gill | Kentucky | H B Smith | Alabama |
| R K Harris | Texas | J W Thompson | Tennessee |
| O L Harky | Tennessee | E D Tilford | Kentucky |
| H F Hamil | Alabama | M Theriot | Louisiana |
| A M Haas | Louisiana | C A Tavel | Tennessee |
| R Inge | Alabama | F L Whitman | Alabama |
| J C Johnson | Tennessee | A A Wheat | Alabama |

Total, 40.

### University of Omaha—Dental Department.

The annual commencement exercises of the Dental Department of the University of Omaha were held in Omaha, Neb., on Tuesday evening, April 4, 1899.

The address to the graduates was delivered by J. B. Meikle.

The degree of D.D.S. was conferred on the following graduates by Rev. David R. Kerr, Ph.D., D.D., chancellor of the university:

| NAME. | RESIDENCE. | NAME. | RESIDENCE. |
|---|---|---|---|
| Cecil E Beebe | Nebraska | Percy J Hunter | Nebraska |
| Zoro D Clark | Nebraska | Geo H Kay | Nebraska |
| Arthur G Greene | Iowa | Fred N Kemp | Missouri |
| Alfred N Hagan | Nebraska | Pauline Koobetsheck | Iowa |
| Charles O Hald | Nebraska | Leslie G Meyers | Nebraska |
| Milton R Hendrix | Nebraska | Chas B Rich | Nebraska |
| Walter J Hostetter | Iowa | John G Somers | Nebraska |

Total 14.

### University of Tennessee, Dental Department.

The annual commencement exercises of the Dental Department of the University of Tennessee were held in Nashville, Tenn., on Tuesday, March 28, 1899.

The charge to the graduates was delivered by Professor John H. DeWitt, A.B., LL.B., and the valedictory by F. A. Blanchard, D.D.S.

The number of matriculates for the session was 104.

The degree of D.D.S. was conferred on the following graduates by Charles W. Dabney, Ph.D., LL.D., president of the university:

| NAME. | RESIDENCE. | NAME. | RESIDENCE. |
|---|---|---|---|
| W H Bailey | Tennessee | W A James | Mississippi |
| Robt M Black | Mississippi | S R Jones | Tennessee |
| F A Blanchard | Louisiana | Wm C King | Tennessee |
| Marion A Bryant | Tennessee | Ernest A Long | Arkansas |
| W M Chambliss | Mississippi | J H McLean | North Carolina |
| Herbert R Clark | Tennessee | Edgar W Moose | North Carolina |
| D B Dawson | Tennessee | R A Nicholson | Mississippi |
| Jas W Dennis | Arkansas | John C Parr | Tennessee |
| C B Fowlkes | Tennessee | J A Pelky | California |
| A W Gould | Tennessee | J B Risenhoover | Kentucky |
| E P Gould | Tennessee | J A Rule | Michigan |
| O L Gould | Tennessee | Baxter C Scott | Mississippi |
| E R Hart | Missouri | J L Ward, M.D | Tennessee |
| Alex H Jackman | Kentucky | C L White | Virginia |

Total, 28.

### University of Buffalo.

Dental Department. Commencement exercises Tuesday evening, April 25, 1899, at 8 o'clock. Music Hall. Graduates:

| | | |
|---|---|---|
| Frank Anderson | Charles E Gillam | Arthur B Muir |
| G Morris Austin | Gladstone Goode | Emanuel Muntz |
| William G Beaumont | Bertrand O Harmon | Daniel J Murray |
| Charles A Bennett | Charles H Hickelton | Thomas F O'Shea |
| Frank J Biker | Abram Hoffman | George O'Leary |
| M Courtney Bradley | George L Horton | Frederick W Orwan |
| William J Burke | Arthur F Isham | Charles L Peck |
| Duncan A Cant | William D Jacobs | Herman E Reynolds |
| Frederic W Champlin | Alfred O Jerrett | William J Roche |
| Charles H Churchill | Arthur J Jessel | Charles W Rowe |
| Arthur B Cobb | Clement D Kennedy | Clare E Robinson |
| Frank W Cook | Albert Kinsella | Robert Sabin |
| Frederic S Cox | Francis M Lee | Robert R Schmidt |
| Leon V Cursons | William N Leonard | H H A Semtner |
| Guy R Danforth | Harry H Luton | Grace N Shirley |
| Joseph A Dixon | Harry K Mason | Howard A Smith |
| George E Dougall | R G W Merkley | Louis W Smith |
| John E Dunn | Stanley A Merkley | Alonzo W Tracy |
| Harry G Fairfield | John E Middaugh | James F Wardner |
| Robert J Fletcher | G Frank Mouthrop | William H Willson |
| Charles J Fraley | Mansfield E Mooney | Herbert W Whitney |

Total 63.

### Kansas City Dental College.

The annual commencement exercises of the Kansas City Dental College were held in Coates Opera House, Kansas City, Mo., on Saturday, April 1, 1899.

The number of matriculates for the session was eighty-five.

The degree of D.D.S. was conferred on the following graduates by Alton H. Thompson, D.D.S., president of the college:

| NAME. | RESIDENCE. | NAME. | RESIDENCE. |
|---|---|---|---|
| D M Austin | Missouri | Harry House | Kansas |
| F H Beaumont | Nebraska | M I Hults | Kansas |
| P J Bentz | Nebraska | O C Haldeman | Missouri |
| C W Bull | Kansas | Henri Letord | Missouri |
| J W Bamber | Kansas | M A Miller | Nebraska |
| W L Caldwell | California | E R Mathers | Nebraska |
| J R Cowdery | Kansas | C A McAntire | Missouri |
| R A Chenoweth | Kansas | C A Martin | Kansas |
| Lloyd Davis | Illinois | R S McKee | Kansas |
| W H Hargis | Missouri | O A Pennock | Illinois |
| F J Hunger | California | W A Swisher | Kansas |
| C E Hocker | Missouri | O D Wells | Kansas |

Total 24.

### Western Dental College.

The ninth annual commencement exercises of the Western Dental College were held in the Auditorium Theater, Kansas City, Mo., on Tuesday afternoon, April 4, 1899.

The annual address was delivered by Hon. James A. Reed, and the faculty address by W. F. Kuhn, M.D.

The degree of D.D.S. was conferred on the following graduates by D. J. McMillen, M.D., D.D.S.:

| NAME. | RESIDENCE. | NAME. | RESIDENCE. |
|---|---|---|---|
| Wm M Bourn | Missouri | Roy W McDonald | Missouri |
| Will A Boyd | Kansas | Chas W Means | Kansas |
| David R Brockman | Washington | R H Merrick | North Dakota |
| Elma J Brockman | Washington | Victor C Nelson | Kansas |
| Frank N Buck | Kansas | John V Prunty | Texas |
| Elvin T Douglass | Missouri | Julian B Reed | Texas |
| Chas C Ehlers | Missouri | Clarence W Sloan | Missouri |
| Albert C Ewart | Canada | John W Smyser | Kansas |
| Chas H Gillham | Missouri | Chas C Sterrett | Kansas |
| John F Grammer | Texas | Geo E Taylor | Kansas |
| Franklin P Hair | Missouri | August A Weber | Kansas |
| Henry T Harvey | Kansas | A L VanArsdall | Missouri |
| F B Henderson | Missouri | Hugh R West | Kansas |
| C E Hereford | Kansas | Herbert J Wetmore | Kansas |
| T Russell Hill | Missouri | Jonas C Whitmer | Missouri |
| Wm C Lampe | Kansas | Edgar P Yarnell | Missouri |
| J Amos Lovell | Missouri | Henry J Zeller | Nebraska |

Total 34.

### Milwaukee Medical College—Dental Department.

The annual commencement exercises of the Dental Department of Milwaukee Medical College were held in the Davidson Theater, Milwaukee, Wis., on Monday, April 3, 1899.

The number of matriculates for the session was one hundred and thirty-five.

The degree of D.D.S. was conferred on the following graduates by G. V. I. Brown, D.D.S., M.D., C.M., dean:

| NAME. | RESIDENCE. | NAME. | RESIDENCE. |
|---|---|---|---|
| Howard C Bright | Wisconsin | Joseph A Krainik | Wisconsin |
| Edward W Bruns | Wisconsin | Conrad H May | Wisconsin |
| Edward J Conley | Wisconsin | John E Purtell | Wisconsin |
| Ezra R Cristman | Wisconsin | Peter A Reuter | Wisconsin |
| James W Dwyer | Minnesota | Herman A Simon | Wisconsin |
| Jas S Danforth | Wisconsin | Henry F Strong | Wisconsin |
| Stephen C Durham | Michigan | David H Thomas | Illinois |
| John J S Freimann | Wisconsin | Arthur N Thompson | Wisconsin |
| Irving H Fowle. | Wisconsin | Chas M Vandenburgh | Wisconsin |
| Leroy H Giffen | Wisconsin | George H Wilde | Illinois |
| Frank W Hamback | Wisconsin | | |

Total 22.

### Baltimore Medical College—Dental Department.

The annual commencement exercises of the Dental Department of Baltimore Medical College were held in the Academy of Music, Baltimore, Md., on Thursday afternoon, April 6, 1899.

The Hippocratic oath was administered to the graduates by Charles G. Hill, M.D. The valedictory address was delivered by Rev. E. T. Lawrence, B.A.

The number of matriculates for the session was seventy-three.

The degree of D.D.S. was conferred on the following graduates by Charles G. Hill, M.D., president of the college:

| NAME. | RESIDENCE. | NAME. | RESIDENCE. |
|---|---|---|---|
| J H Ammenheuser | Maryland | Emanuel Lehmann | New York |
| Heber B Beadle | Connecticut | Robert C Lewis | Virginia |
| Chas E Culver | Pennsylvania | John F Mayock | Pennsylvania |
| Jas W S Dickison | Canada | J F Miller | New York |
| Benj L Fisk | New York | H S Simmons | New Brunswick |
| Herman R Hall | Maine | John E Swallow | Maryland |
| Ralph Lange | Maryland | Michael H Toomey | Massachusetts |

Total 14.

### Central College of Dentistry.

The annual commencement exercises of the Central College of Dentistry were held in Plymouth Church, Indianapolis, Ind., on Tuesday, April 11, 1899.

The number of matriculates for the session was fifty-four.

The degree of D.D.S. was conferred on the following graduates by Dr. J. E. Cravens, president of the college: Charles Bamhill, Frank Terrill, William Card, Herbert Stomcifer, all of Indiana.

### Birmingham Dental College.

The annual commencement exercises of the Birmingham Dental College were held in O'Brien's Opera House, Birmingham, Ala., on March 31, 1899, at 8:30 P. M.

The address to the graduating class was delivered by Hon. Joseph H. Merrill, of Thomasville, Ga.

The number of matriculates for the session was forty-two.

The degree of D.D.S. was conferred upon the following graduates by T. M. Allen, D.D.S., dean:

| NAME. | RESIDENCE. | NAME. | RESIDENCE. |
|---|---|---|---|
| W D Carmichael | Wisconsin | B M Ohine | Florida |
| J S Daniel | Alabama | J W Ried | Alabama |
| R S Hunt | Alabama | Ira Williamson | Mississippi |
| W H Marsh | Alabama | | |

### Illinois School of Dentistry.

Graduates for 1899:

| | | |
|---|---|---|
| Harry C Snyder | E B Barrow | Hanferd Browne |
| Mac Tilton | W A Rausch | J B Zielinsky |
| M C Hoag | Jete L McCarthy | W R McGarvey |
| R W Dodez | H Brophy | H F Grantvedt |
| T F Condit | W B Spafford | Chas H Wambold |
| E M Carter | J Addison Brown | W C Shallanberger |
| A C Aldrich | W L Bradford | N J Hendricks |
| Claude E Frazier | George Umbenhaur | H N Lancaster |
| O P Jesse | Fred C Allender | |

Total 26.

### University of Omaha.

Dental Department. Graduates, 1899:

| NAME. | RESIDENCE. | NAME. | RESIDENCE. |
|---|---|---|---|
| Cecil Edwin Beebee | Nebraska | Percy James Hunter | Nebraska |
| Zoro Dennis Clark | Nebraska | George Henry Kay | Nebraska |
| Arthur George Greene | Iowa | Fred Norton Kemp | Missouri |
| Alfred Newton Hagan | Nebraska | Pauline Koobetsheck | Iowa |
| Charles Oliver Hald | Nebraska | Leslie Grant Meyers | Nebraska |
| Milton Rieman Hendrix | Nebraska | Charles Bushnell Rich | Nebraska |
| Walter J Hostetter | Iowa | John Gerald Somers | Nebraska |

Total 14.

